# Hypoxia Preconditioned Serum (HPS)-Hydrogel Can Accelerate Dermal Wound Healing in Mice—An In Vivo Pilot Study

**DOI:** 10.3390/biomedicines10010176

**Published:** 2022-01-14

**Authors:** Jun Jiang, Ursula Kraneburg, Ulf Dornseifer, Arndt F. Schilling, Ektoras Hadjipanayi, Hans-Günther Machens, Philipp Moog

**Affiliations:** 1 Experimental Plastic Surgery, Clinic for Plastic, Reconstructive and Hand Surgery, Klinikum Rechts der Isar, Technische Universität München, D-81675 Munich, Germany; junqing.jiang@mri.tum.de (J.J.); Ursula.Kraneburg@mri.tum.de (U.K.); e.hadjipanayi@googlemail.com (E.H.); 2 Department of Plastic, Reconstructive and Aesthetic Surgery, Isar Klinikum, D-80331 Munich, Germany; ulf.dornseifer@isarklinikum.de; 3 Department of Trauma Surgery, Orthopedics and Plastic Surgery, Universitätsmedizin Göttingen, D-37075 Gottingen, Germany; arndt.schilling@med.uni-goettingen.de

**Keywords:** peripheral blood cells, blood-derived therapy, hypoxia, angiogenesis, hypoxia preconditioned plasma, hypoxia preconditioned serum, lymphangiogenesis, lymphatic regeneration, wound healing

## Abstract

The ability to use the body’s resources to promote wound repair is increasingly becoming an interesting area of regenerative medicine research. Here, we tested the effect of topical application of blood-derived hypoxia preconditioned serum (HPS) on wound healing in a murine wound model. Alginate hydrogels loaded with two different HPS concentrations (10 and 40%) were applied topically on full-thickness wounds created on the back of immunocompromised mice. We achieved a significant dose-dependent wound area reduction after 5 days in HPS-treated groups compared with no treatment (NT). On average, both HPS-10% and HPS-40% -treated wounds healed 1.4 days faster than NT. Healed tissue samples were investigated on post-operative day 15 (POD 15) by immunohistology and showed an increase in lymphatic vessels (LYVE-1) up to 45% with HPS-40% application, while at this stage, vascularization (CD31) was comparable in the HPS-treated and NT groups. Furthermore, the expression of proliferation marker Ki67 was greater on POD 15 in the NT-group compared to HPS-treated groups, in accordance with the earlier completion of wound healing observed in the latter. Collagen deposition was similar in all groups, indicating lack of scar tissue hypertrophy as a result of HPS-hydrogel treatment. These findings show that topical HPS application is safe and can accelerate dermal wound healing in mice.

## 1. Introduction

The demographic shift towards an older society, combined with an increase in comorbidities such as diabetes or cardiovascular diseases, has led to an increasing prevalence of chronic wounds, which have become not only a medical issue but also a significant economic burden, consuming 2–4% of health care budget worldwide [1]. It is estimated that 1 to 2% of the population will suffer from chronic wounds during their lifetime in industrialized countries [2]. In the US alone, 3 to 6 million people suffer from non-healing wounds [3], with an overall cost to the American healthcare system of around three billion dollars a year [4,5]. Worldwide, the costs are estimated up to 25 billion dollars per year [6]. In Germany, around 1 million citizens suffer from chronic wounds (as of 2012) [7], which can lead to social isolation, long-term inability to work and loss of quality of life [8].

As we come to understand wound healing processes in greater depth, we now acknowledge that this is a complex system of biological interactions that requires the coordination of several cell types, intra- and extracellular mechanisms and signaling pathways. Either defect, loss, or dominance of any factor in these interactions can lead to failure of the entire system, resulting in chronic wounds [9]. New wound healing approaches that are aimed towards improving these interactions must be delivered in a targeted and sustainable manner to effectively modulate these complex mechanisms [10]. Due to the significant risks of systemic toxicity and numerous side effects of systemic drug administration, therapeutic strategies are increasingly focusing on locally-targeted application [11]. The use of topical approaches improves bioavailability and facilitates the achievement of controlled drug concentration levels within a safe therapeutic range [12].

Current strategies for promoting angiogenesis in acute and chronic wounds are frequently based on the exogenous supply of recombinant angiogenesis factors [13]. However, the concept of employing the body’s own natural resources towards regeneration has recently gained increased interest [14]. Our preferred approach focuses on utilizing a physiologically-adjusted growth factor therapy which can be administered using a minimally-invasive method [15,16]. We have shown that the localized delivery of peripheral blood-derived hypoxia-induced growth factor-mixtures can serve as a tool for the natural promotion of spatio-temporally controlled regeneration [15,16,17,18]. In accordance to the principle that the host response for optimal wound healing depends on multiple regulatory pathways [19,20], these paracrine proteins are used to stimulate a range of targeted cellular responses including wound angiogenesis, lymphangiogenesis, matrix deposition and re-epithelialization through fibroblast migration and proliferation [16,18,20,21,22,23,24,25]. Previous work by our group has already shown that collagen scaffolds containing hypoxia-induced growth factor proteins, produced by dermal fibroblasts, can promote vascularization and improve deep scaffold oxygenation when implanted in vivo in a rabbit model [26,27]. In contrast to dermal fibroblasts, however, that must be obtained through an excisional biopsy, peripheral blood cells (PBC, or more specifically peripheral mononuclear cells, PBMC) readily serve as growth factor providers, as they exhibit several advantages: there is, firstly, abundant supply, they are easy to obtain from peripheral venous blood and, if used autologously, there is no risk of rejection by the immune system [16,17]. PBCs react to stress (hypoxia, ischemia, inflammation, ultrasound, etc.) with an upregulation of angiogenic growth factors such as VEGF [16,18,23,28,29,30,31], bFGF [16,18,23,29,30], IL-8 [16,18,23,30], MMP-9 [16,18,23,30], as well as the downregulation of antiangiogenic factors such as TSP-1 [16,17,18,23].

Based on these fundamental concepts, we had previously developed a novel approach for providing a biomimetic cocktail of blood-derived growth factor proteins within a locally-deployable carrier [16,17,18]. For this purpose, the primary stimulus of the wound healing angiogenic response, i.e., hypoxia, is used to stimulate PBCs to produce ‘new’ angiogenic and lymphangiogenic protein factors [16,18,23,24,25]. To obtain these complex growth factor mixtures we utilize a method of hypoxia-adjusted in vitro preconditioning, by cultivating PBCs ex vivo within a self-regulated low-oxygen microenvironment [16,17,21]. Instead of using a hypoxic incubator chamber, we allow cell-mediated O_2_ consumption to automatically generate local pericellular (i.e., surrounding the cell layer) hypoxia (~1% O_2_) within the blood-containing chamber. Since blood cells sediment in the course of hypoxic incubation, protein factors are secreted/released into the serum (hypoxia preconditioned serum: HPS) and can be separated from PBCs through filtration without the need for centrifugation, as currently required in the platelet-rich plasma (PRP) method [16,18,23,32].

Several in vitro experiments have validated the effectiveness of this approach in promoting an angiogenic response in PBCs. Pro-angiogenic (VEGF) factor levels were upregulated with longer incubation time and peak at 4 days of incubation [16,18,23]. In HUVEC (human umbilical vein endothelial cell)-sprouting assays and aortic ring assays, HPS stimulated a greater invasion of endothelial cells, more vascular sprouts (sprouting angiogenesis) and greater sprout length compared to the recombinant VEGF- and PRP-treated groups [18,23]. Regarding lymphangiogenesis, HPS has been tested in a ductus thoracicus sprouting assay and exhibited superior formation of new lymphatic vessels after 4 days compared to other lymphatic stimulants such as FCS 20% [25]. Importantly, we have shown that there was no loss of pro-angiogenic activity in vitro when blood was derived from patients who receive oral anticoagulation due to underlying vascular pathology or those who suffer from diabetes mellitus type 1 and type 2 [22]. Thus, patients with arteriosclerosis and diabetic micro-angiopathies, who do have a higher prevalence of chronic wounds, could potentially benefit from HPS therapy.

The controlled delivery of such complex, yet physiological growth factor mixtures in vivo could represent a solution for overcoming the limited ability of chronically damaged tissue to optimally switch on angiogenic and lymphangiogenic responses, which are essential drivers of wound regeneration. In this context, we previously engineered an integral HPS-hydrogel system that sequesters the protein factors at the site of application, allowing the gradual generation of spatiotemporal gradients, which are essential for the development of directionally-controlled angiogenesis [15,16,18]. Hydrogel carriers are easy to clinically apply, either through injection or topical application and also have the advantage of potentially serving as scaffolds for cellular ingrowth and biological defect coverage [15,16,17,18]. In this study, we sought out to demonstrate the effects of HPS in an in vivo murine excisional wound model, in which a silicon splint is used to prevent wound contraction, thus, allowing the wounds to heal solely through epithelialization [33]. Here, we used immunocompromised athymic nude mice to investigate the effects of human blood-derived HPS, loaded onto an alginate hydrogel carrier, with regards to the time to full wound closure, assessed by digital image analysis and immunohistochemical staining for vascularization (CD31), lymphangiogenesis (LYVE-1) and proliferation activity (Ki-67). We also measured collagen and connective tissue generation through Masson-Trichrom staining. Our findings confirm the safety, but also demonstrate the efficacy of HPS treatment in murine wound healing, thus offering a platform for potentially engineering a therapy for human application.

## 2. Materials and Methods

### 2.1. Ethical Approval

All in vivo experiments were performed under the guidance of Zentrum für Präklinische Forschung (ZPF) Veterinary Department of the Technical University of Munich, Germany and were approved by the General Administration of the Free State of Bavaria (ROB-55.2-2532.Vet_02-17-189; date of approval: 21 September 2018). All blood donors provided written informed consent as directed by the ethics committee of the Technical University Munich, Germany, which approved this study (File Nr.: 104/21 S-EB; date of approval: 19 February 2021).

### 2.2. Production of Hypoxia Preconditioned Serum (HPS) and HPS-Hydrogel

HPS was produced following the protocol described previously by Hadjipanayi et al. [18]. Briefly, peripheral venous blood was drawn under sterile conditions from 3 blood donors and collected into separate 30 mL polypropylene syringe (Omnifix^®^, B Braun AG, Melsungen, Germany) for HPS preparation. Five milliliters of air was drawn into the syringe through a 0.2 µm filter (Sterifix^®^, B Braun AG, Melsungen, Germany), with the plunger fully retracted. Subsequently, the syringes were placed upright in the incubator (37 °C/5% CO_2_) and incubated for 4 days. Following incubation, the blood was separated through sedimentation into three layers (from top to bottom: serum, clot, red blood cell layer), so that the top layer (HPS) could be filtered (Sterifix^®^, B Braun AG, Melsungen, Germany) into a new syringe, removing cells/cellular debris. In the next step, the HPS of the 3 blood donors was pooled. As a vehicle for controlled delivery of the HPS protein factors, we mixed the HPS at 10 and 40% final concentration with a clinically available alginate hydrogel (Nu-Gel, KCI GmbH, Wiesbaden, Germany) to achieve a topical application for wounds. HPS-Hydrogel was stored at 4 °C until testing.

### 2.3. Animals

Six-week-old female athymic nude mice *Crl:NU(NCr)-Foxn1nu* were purchased from Charles River laboratories (Wilmington, MA, USA), which have a spontaneous deletion in the *Foxn1* gene that causes a deteriorated or absent thymus. This results in an inhibited immune system with reduction of number of T-cells which leads to an impaired immune system of the mice. The athymic nude mouse is valuable for research since it can be applied with many different types of tissue and cells, including those of human origin, as there is no immune response to them. Animals were housed five animals per cage prior to surgery and alone post procedure in a temperature-controlled animal facility with a 12-h light/dark cycle. The mice were acclimated to their environment for 10 days prior to the procedure and were allowed food and water ad libitum.

### 2.4. Full-Thickness Excisional Wounds

Splinted full-thickness excisional wounds were created following the model described by Galiano et al. [33]. Anesthesia was induced with isoflurane 5%, 1 L/min oxygen and maintained at isoflurane 1–3%, 1 L/min oxygen. The mouse was placed on a heating mat to prevent a drop of body temperature during surgical procedure. The dorsum of each mouse was cleaned with antiseptics (Octenisept, Schülke & Mayr GmbH, Norderstedt, Germany) and two identical 8 mm circular wounds were created on each side of the dorsum at the level of the shoulder with a sterile 8 mm punch biopsy tool. Surgical forceps were used to lift off the skin in the center of the outline and a piece of tissue extending through the subcutaneous tissue including the panniculus carnosus was excised with microsissors. 0.5 mm thick donut shaped silicone splints with 8 mm inner diameter were fixed to the surrounding wound edge using an immediate bonding cyano-acrylate adhesive (UHU 46971, UHU GmbH & Co. KG, Bühl, Germany) and simple interrupted 6-0 nylon sutures (Ethicon Inc., Raritan, NJ, USA). Thus, the falsification of results by wound contraction of the panniculus carnosus region was avoided. For the treatment groups, HPS-10% or HPS-40% was used and was compared to wounds in which sterile saline was placed in the wound bed (no treatment = NT). Wounds were covered with clear occlusive dressing (KCI GmbH, Wiesbaden, Germany), which was changed on alternating days. Upon completion of surgery, the mice were placed in separate mice cages and observed until they fully recovered from anesthesia. We used 8 mice for each of the HPS-10%, HPS-40% and NT group, totaling a number of 16 wounds per group.

### 2.5. Wound Analysis

Digital photographs were taken on the day of surgery and on post-operative days (POD) 3, 5, 7, 9, 11, 13 and 15. Time to closure was defined as the time period at which the wound bed was completely epithelialized, as blindly assessed by three independent reviewers. Wound area was analyzed by tracing the wound margin and calculating the area (in mm^2^) using ImageJ software (NIH, Bethesda, MD, USA, version 1.53) [34]. As the splint has a constant and predefined area, it was used to normalize and calculate the wound sizes. Images were analyzed by three blinded observers. A wound was considered completely closed when the wound area was macroscopically no longer visible.

### 2.6. Staining and Immunohistochemistry

Mice were sacrificed under 5% isoflurane and cervical dislocation. The wound sites were harvested by excising them in the shape of the inner circle of the silicone ring to the subdermis with microscissors. The round-shaped tissue samples were bisected into identical halves and were immediately fixed in 4% paraformaldehyde at 4°C overnight. Samples were dehydrated in series of ethanol, embedded in paraffin and serially cut from the center into sections of 4-μm thickness, examined on a coated slide glass and were stained with H&E and Masson-Goldner’s Trichrom. Immunohistochemical staining for endothelial cell marker CD31 (dianova GmbH, Hamburg, Germany), lymph-endothelial cell marker LYVE-1 (Abcam, Cambridge, UK) and proliferation marker Ki-67 (Abcam, Cambridge, UK) were performed on the fully automated Bond-Max system (Leica Biosystems, Nussloch, Germany). All immunohistochemical antibodies were followed by the two-step peroxidase technique and the staining process was achieved using diaminobenzidine (DAB) chromogen. A Leica Aperio microscope (Leica Biosystems, Nussloch, Germany) was used to digitally scan the slides. Masson-Goldner’s Trichrom staining and immunohistochemistry slides were analyzed following the method used by Mezei et al. [35]. Using ImageJ Hue/Saturation/Brightness color filtering we could measure the area of a given tissue marked by collagen staining or DAB. Briefly, the following settings were used (numbers indicate minimum and maximum values while letters in brackets indicate filter type: P—pass; S—stop): DAB Hue 44/255 (S), Saturation 37/255 (P), Brightness 0/255 (P); Trichrom Hue 0/216 (P), Saturation 56/255 (P), Brightness 0/255 (P). A high-powered field (HPF) was selected at 2500 × 2500 pixels and each tissue sample was analyzed by four separate HPFs. By color filtering, DAB and collagen staining marked areas were calculated as pixels. The epidermal thickness was determined using the H&E sections. Measurements and quantification were performed with Aperio ImageScope (Leica Biosystems, Nussloch, Germany) by evaluating eight random HPFs of each tissue sample.

### 2.7. Statistical Analysis

Data sets were analyzed by one-way analysis of variance (ANOVA), with subsequent comparisons using Tukey’s post-hoc analysis, when one independent variable is present. In the case of repeated measures with two independent variables, a two-way repeated measures ANOVA with Tukey’s multiple comparisons test was applied. All values are expressed as means ± standard error of the mean (SEM). A value of *p* < 0.05 was considered statistically significant (* *p* < 0.05, ** *p* < 0.01, *** *p* < 0.001, **** *p* < 0.0001).

## 3. Results

### 3.1. HPS Accellerates Wound Closure

Using two different HPS concentrations, we sought to measure dose-dependent effects on wound healing in mice. We tested a low (10%) and high (40%) concentration HPS-hydrogel formulation on a murine excisional wound model (note: there was a loss of 5 mice due to anesthesiology complications on the day of surgery). No HPS-related complications (e.g., infection or bleeding, autoimmune reaction) occurred in the duration of the study; thus, the following treatment groups were included in this experiment: HPS- 10% and 40% with 7 mice (14 wounds) each and no treatment (NT) group (rinsed with sterile saline solution) with 5 mice (10 wounds). We performed a dressing change with re-application of HPS-hydrogel and took wound photographs on post-operative day (POD) 3 and thereafter every 2 days until POD 15.

**Figure 1:**
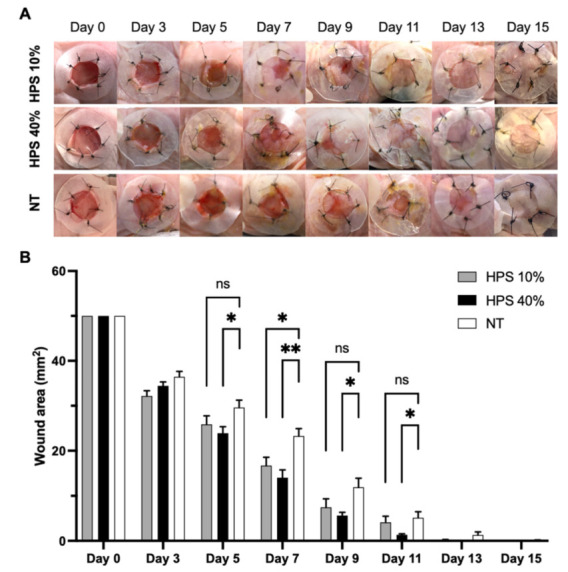
Accelerated wound healing in HPS-10% and 40% treated wounds. (**A**) Representative macroscopic photographs of full-thickness wounds in the excisional murine skin model on post-operative day (POD) 0, 3, 5, 7, 9, 11, 13 and 15. Two treatment groups were tested, HPS-10% (*n* = 14) and HPS-40% (*n* = 14) vs. no treatment (NT) (*n* = 10). (**B**) Plot showing wound surface area (50 mm^2^ wound area on POD 0) change from POD 0 to POD 15 in the HPS-10%/-40% and NT groups. HPS-40% group has smaller wound area compared to NT already at POD 5 and POD 7 (*p* = 0.04 and *p* = 0.002 respectively), HPS 10% group has smaller wound area compared to NT at POD 7 (*p* = 0.03). Wound healing kinetics of HPS-10% and -40% treatment groups showed to be significantly better than NT (*p* = 0.02 and *p* = 0.0002, respectively). Two-way repeated measures ANOVA with Tukey’s multiple comparisons test. Data points represent means ± SEM. * = *p* < 0.05, ** = *p* < 0.01, ns = non-significant.

The wounds were determined as completely healed in a mean of 11.7 days in both the HPS-10% (11.71 ± 0.28 days) and HPS-40% (11.67 ± 0.32 days) group, compared to 13.07 ± 0.57 days in the NT group, thus showing a faster wound healing response by 1.4 days (*p* < 0.05) (Figure 1). Although both HPS-hydrogel treated groups achieved full wound closure at approximately the same timepoint, HPS-40% treated wounds appeared to heal relatively faster than HPS-10%: the wound surface area was already significantly smaller at day 5 by 19.2% compared to NT (23.91 ± 1.45 cm^2^ vs. 29.60 ± 1.66 cm^2^) (*p* = 0.04) and by 39.8% at day 7 (14.00 ± 1.73 cm^2^ vs. 23.28 ± 1.63 cm^2^) (*p* = 0.002), while in the HPS-10% group wound surface area was only significantly reduced by 28.3% at day 7 (16.69 ± 1.86 cm^2^ vs. 23.28 ± 1.63 cm^2^) (*p* = 0.03). However, with regards to wound healing kinetics of the total time course, treatment with HPS-10% showed to be significantly better compared to NT (*p* = 0.02), whereas HPS-40% exhibited even superior performance (vs. NT; *p* = 0.0002). On the other hand, a true significant difference between HPS-10% and -40%-groups was not detected at any time point, although mean wound areas of HPS-40% were smaller at every POD. Thus, we observed a relative benefit with higher HPS concentration regarding earlier wound area reduction.

### 3.2. Effect of HPS Treatment on Wound Vascularization

**Figure 2:**
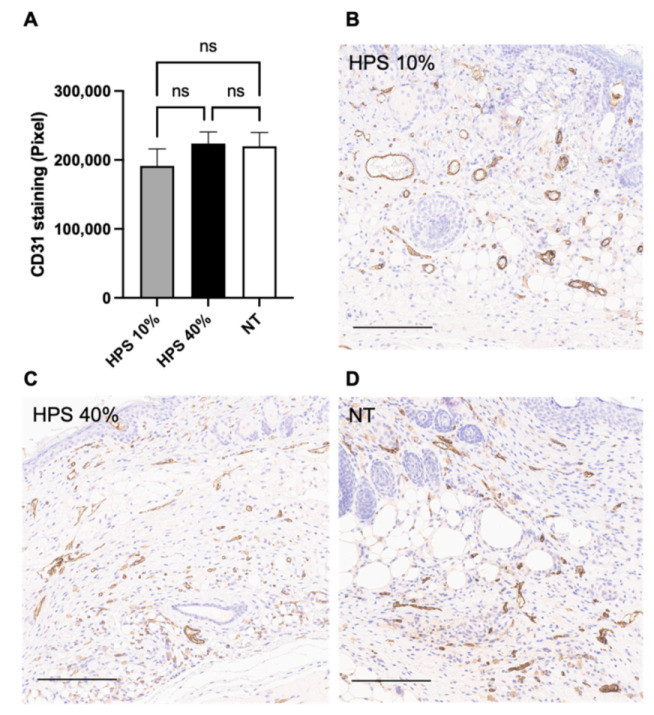
CD31 DAB Immunostaining. (**A**) Quantitative measurement of CD31 Diaminobenzidine (DAB) staining in HPS-10% (*n* = 14) and 40% (*n* = 14) treated murine wounds vs. no treatment (NT) (*n* = 10) on POD 15 by color segmentation of DAB-positive cells calculated as pixels. Vascularization did not appear different in either HPS-treated group compared to the NT group. Data are means ± SEM. Ns = non-significant. One-way ANOVA with Tukey’s post-hoc test. (**B**–**D**) Representative high-power fields of CD31 DAB Immunostaining of HPS-10%, HPS-40% -treated wounds and NT wounds. Scale bar = 200 μm.

Immunohistochemistry (IHC) using diaminobenzidine (DAB)-dye was carried out on POD 15 in healed tissue samples to analyze angiogenesis with CD31 staining. CD31 is highly expressed on the surface of endothelial cells and is involved in cell-cell associations during the formation of new capillaries [36]. ImageJ software was used with semiautomatic color segmentation to measure the extend of DAB chromogenic reaction of CD31 stained proteins. For the examined time point, vascularization did not appear different in either HPS-treated group compared to the NT group, showing similar capillary density and vessel size in the subcutaneous layer at the stage of fully healed wounds (Figure 2). Given the observed accelerated wound closure in the HPS-treated groups, this data suggests that angiogenesis here may have been completed much earlier than POD 15 and probably even before POD 13, when differences in wound surface area were no longer statistically different compared to the NT group.

### 3.3. HPS Promotes Lymphangiogenesis

**Figure 3:**
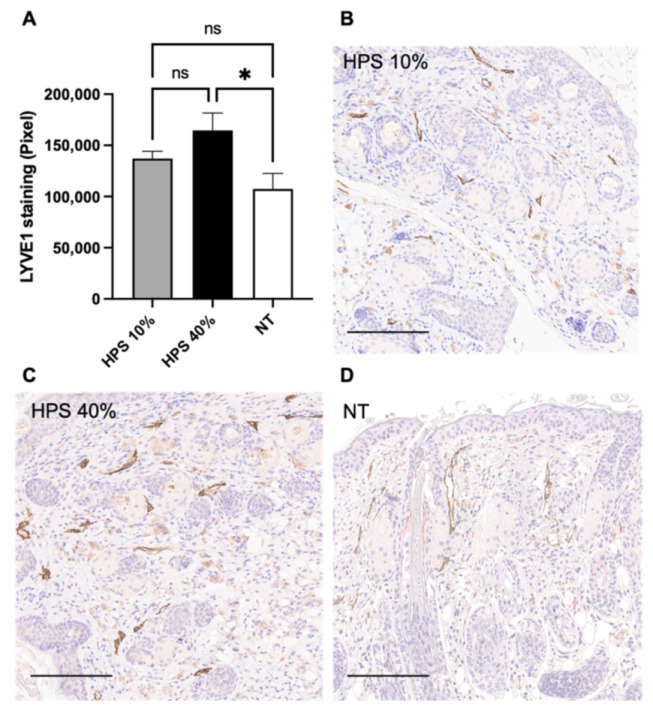
LYVE-1 DAB Immunostaining. (**A**) Quantitative measurement of lymphatic vessel endothelial hyaluronic acid receptor (LYVE-1) Diaminobenzidine (DAB) staining in HPS-10% (*n* = 14) and 40% (*n* = 14) treated murine wounds vs. no treatment (NT) (*n* = 10) on POD 15 by color segmentation of DAB-positive cells calculated as pixels. HPS-40% showed higher LYVE-1 expression compared to the NT group (*p* = 0.01). Data are means ± SEM. * = *p* < 0.05, ns = non-significant. One-way ANOVA with Tukey’s post-hoc test. (**B**–**D**) Representative high-power fields of LYVE-1 DAB Immunostaining of HPS-10%, HPS-40% -treated wounds and NT wounds. Scale bar = 200 μm.

Fully healed wounds on POD 15 were further investigated for lymphatic vessel endothelial hyaluronic acid receptor (LYVE-1) expression using DAB-IHC. LYVE-1 is a lymphatic-specific marker, which is expressed on both sides of lymphatic endothelial cells and is involved in lymph node homing of leukocytes [37]. LYVE-1 was augmented in the subcutaneous layer of HPS-40% treated wounds compared to NT (*p* = 0.01), indicating superior lymphangiogenesis with a higher concentration of HPS application (Figure 3). Mean LYVE-1 expression in the HPS-40% group was up to 45% higher than NT (164656 vs. 113218 Pixels). Mean LYVE-1 expression in the HPS-10% group was also higher than NT, but this difference was not significant (*p* = 0.65).

### 3.4. Reduced Cell Proliferation in HPS-10%-Treated Wounds

**Figure 4:**
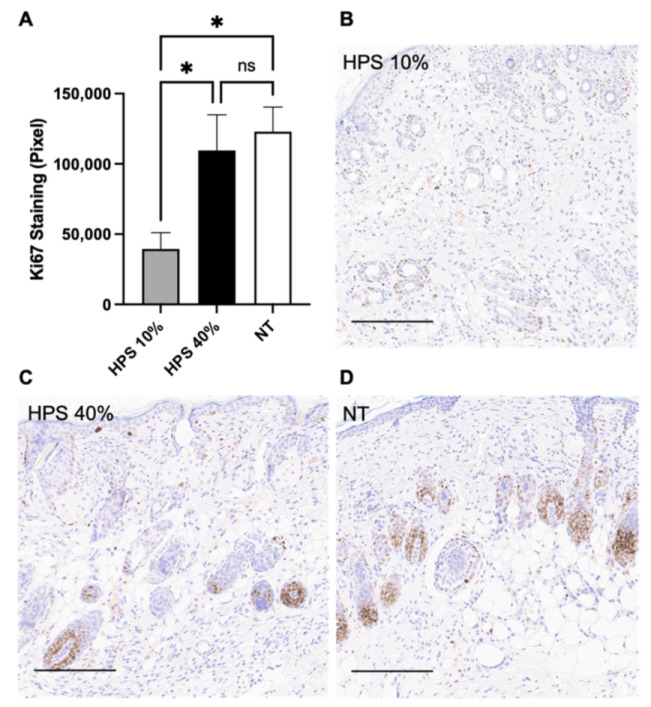
Ki67 DAB Immunostaining. (**A**) Quantitative measurement of Ki67 Diaminobenzidine (DAB) staining in HPS-10% (*n* = 14) and 40% (*n* = 14) treated murine wounds vs. no treatment (NT) (*n* = 10) on POD 15 by color segmentation of DAB-positive cells calculated as pixels. HPS-10% group showed the least Ki67 expression compared to HPS-40% (*p* = 0.03) and NT group (*p* = 0.02). Data are means ± SEM. * = *p* < 0.05, ns = non-significant. One-way ANOVA with Tukey’s post-hoc test. (**B**–**D**) Representative high-power fields of Ki67 DAB Immunostaining of HPS-10%, HPS-40% treated wounds and NT wounds. Ki67 positive cells were mainly located on POD 15 in the dermal papillary cells as well as in the basal and suprabasal epithelial layers. Scale bar = 200 μm.

Cell proliferation by Ki67 was then analyzed to investigate the regeneration speed of fully epithelialized tissue on POD 15. Ki67 is an antigen which is expressed in the G1, G2, S and M phases of the cell cycle, but not in resting cells of G0 phase [38]. Here, Ki67 using DAB-IHC was stained especially in keratocytes in the epidermis and mainly in the basal and suprabasal layers as well as dermal papillary cells. The highest level was measured in the NT group (*p* = 0.02) compared to HPS-10% (Figure 4). Furthermore, Ki67 staining of HPS-40%-treated tissue was also significantly higher than in the HPS-10% group (*p* = 0.03). There was no difference in Ki67 expression between HPS 40% group and NT (*p =* 0.88). In summary, Ki67 was detected least in the HPS-10% group, indicating that the regeneration process may have been completed earlier here than in the HPS-40% group and NT.

### 3.5. Normal Production of Collagen in HPS-Treated Wounds

**Figure 5:**
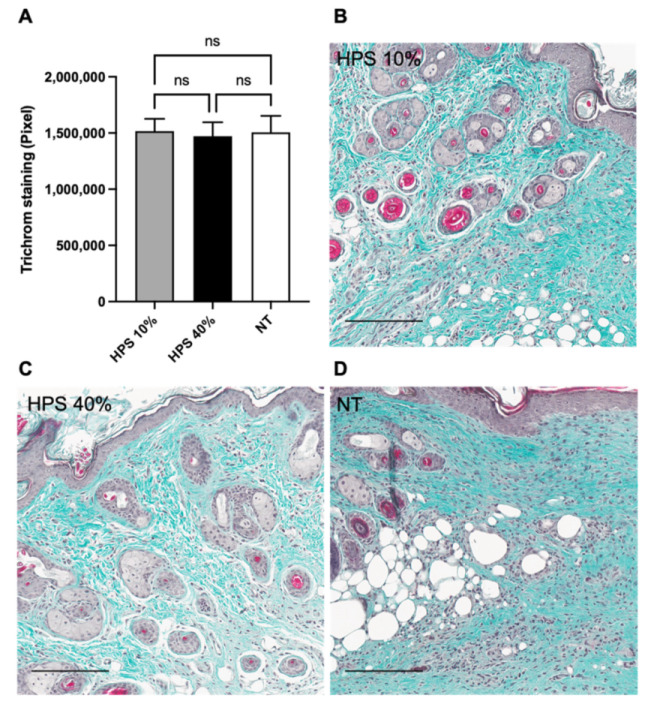
Masson-Goldner-Trichrom staining. (**A**) Quantitative measurement of collagen fiber deposits in the subdermal layers of HPS-10% (*n* = 14) and 40% (*n* = 14) treated murine wounds vs. no treatment (NT) (*n* = 10) on POD 15 by color segmentation of collagen-staining, calculated as pixels. There was no overproduction of collagen in HPS-treated groups compared to NT. Data are means ± SEM. Ns = non-significant. One-way ANOVA with Tukey’s post-hoc test. (**B**–**D**) Representative high-power fields of Masson-Goldner-Trichrom staining of HPS-10%, HPS-40%-treated wounds and NT wounds. Scale bar = 200 μm.

The analysis of connective tissue regeneration was studied by employing Masson-Goldner’s Trichrome staining. Measurements were taken of the green dye of collagen staining by Masson-Goldner’s Trichrom staining procedure following the ImageJ-software method by Mezei et al. [35]. Throughout every tissue sample, there was no overproduction of collagen in HPS-treated groups compared to NT, thus indicating no hypertrophy of scar tissue formation (Figure 5). Furthermore, the return of skin appendages (hair follicles and sebaceous glands) seemed to be equally distributed by gross visualization throughout all healed tissues. The epidermis thickness was determined by software measurement throughout the healed tissue, with no significant differences between treatment and no treatment groups (mean thickness ± SEM: HPS-10% 29.19 ± 1.52 µm, HPS-40% 27.46 ± 1.39 µm, NT 26.62 ± 1.23 µm).

## 4. Discussion

Blood-derived growth factor-based therapies have gradually gathered more attention for their potential use in the regenerative medicine field [14,39]. Our approach focuses primarily on locally delivering a physiological mixture of blood-derived growth factor proteins to the injury site [16,18], which may offer a simple and convenient way to support and accelerate wound regeneration. Here, we tested the regenerative effects of a HPS-loaded alginate hydrogel in an in vivo splinted murine wound model. Using a topical application, we showed faster wound closure by up to 1.4 days compared with no treatment (NT), as well as significant wound surface area reduction by 19.2% starting at day 5 with HPS-40% application. Wound area reduction was accelerated between day 5 and day 11 with HPS application, exhibiting up to 74% smaller wound surface area on day 11 compared with NT (Figure 1B).

The absolute difference between HPS-hydrogel and NT in wound healing kinetics of 1.4 days appears small at first glance. However, it is important to note that the mouse model employed has a high basal wound healing speed which lies in the faster epidermal cell turnover of murine skin (8–10 days vs. 40–56 days in humans) and higher stem cell mitosis [40]. In fact, as murine wound healing is faster than in other wound models (e.g., rabbit, porcine) [41], an increase of 1.4 days is more than 10% of the wound-closure time. Accordingly, a comparable increase in human wounds would shorten the healing time by 5–6 days. More particularly, the splinted murine wound model has been used in several other regenerative wound studies [42,43,44], which also looked at the effects of adipose-derived stem cells (ADSCs) and platelet-rich plasma (PRP), currently the best-known blood-based therapy [39]. In comparison to HPS, growth factor release in PRP relies only on the activation of platelets, that are concentrated through centrifugation to a supraphysiological level [23,45]. As mentioned earlier, HPS does not only comprise platelet-derived factors that are released during blood clotting, but also the complete secretome of growth factor proteins that are produced during the inflammatory and angiogenic/proliferative phases by PBCs [18,23]. Treatment with PRP-only has been shown elsewhere to promote faster wound closure by 0.5 days in the same wound model (even with 60% smaller surgical wound area: 20 mm^2^ vs. 50 mm^2^), whereas the addition of ADSCs to PRP exhibited an acceleration of up to 2.2 days compared to NT [44]. Indeed, a head-to-head comparison of the effects of HPS and PRP in a standardized in vivo wound model remains to be investigated.

The therapeutic approach presented here demonstrates the safety of using human HPS in a xenogeneic murine wound model. In continuation of our work for the safety characterization of HPS-1% and HPS-5% [18], we decided to choose a 10 and 40% mix ratio to exclude adverse effects, such as inflammatory responses, which may be caused by inflammatory cytokines (e.g., IL-8), contained in HPS [16,18,23]. Having also previously examined the in vivo biocompatibility and regenerative effects of HPS-hydrogel on chronic leg ulcers (patient case report) [18], the mix ratios have been adapted to investigate its effects in this standardized wound model. In particular, given that our previous data showed dose-dependency in in vitro angiogenesis experiments [23,25], we aimed to elucidate any similar effects in this model by applying a four-fold dilution ratio. Despite the fact that there was no significant difference in wound healing kinetics between HPS-10% and -40% dosing, we measured smaller mean wound sizes with HPS-40% treatment on every POD (Figure 1B), which indicates greater acceleration of wound healing with a higher HPS concentration, especially in early stages of wound repair.

Interestingly, we found no hypervascularization in HPS-hydrogel treated wounds at the healed stage on POD 15 in comparison to NT (Figure 2). Other studies using the same murine model, in which wounds were treated with MSCs (mesenchymal stem cells) [43] or ADSCs [42], had found significantly elevated CD31 expression in healed tissue around POD 14. In the scope of the observed acceleration of wound surface area reduction, as well as our previous in vitro results [16,18,23], we hypothesize that HPS-hydrogel treatment also promotes angiogenesis, the primary driver of wound healing. However, this response may have been completed much earlier than POD 15. It is also possible that HPS provides regulatory signaling through anti-angiogenic growth factors (e.g., TSP-1, PF-4) which could inhibit excessive blood vessel formation in vivo [18,20,23,25]. This may well be a naturally-occurring adaptive response, to prevent aberrant angiogenesis and vascular leakage once wound healing has been completed - a result that would support the safety of this therapeutic approach. Nevertheless, the exact mechanism(s) through which HPS protein factors may exert these regulatory effects remain unclear.

Beside blood vessel angiogenesis, lymphatic vessel generation plays a critical role in interstitial fluid transport, which reduces excessive oedema and supports angiogenic, inflammatory and proliferative processes, thus contributing to more efficient regeneration [46]. Here, we have shown dose-dependent promotion of up to 45% more lymphatic vessels with HPS-treatment compared to NT (Figure 3). Provided that there was no significant difference in terms of wound surface area between these groups after POD 13, the observation of these differences at POD 15 indicates that lymphangiogenesis may occur at a later stage compared to angiogenesis. We do acknowledge that we investigated a broader overview of the first 15 days post-wounding and that the cellular dynamics and processes, especially regarding angiogenesis and lymphangiogenesis, involved within and after this time-window, have not been closely examined. For this purpose, we aim to conduct further research in a larger animal model (e.g., swine) to develop a better understanding of these spatio-temporal events.

As a marker of cell proliferation and metabolism, Ki67 has been previously investigated on earlier stages of wound healing (POD 6 and 12) in a study of diabetic rat leg ulcers, in which MSCs encapsulated in polysaccharide hydrogels were applied as treatment [47]. It was shown that Ki67-expression was upregulated in the MSC-therapy group compared to NT in the course of active wound repair. In our study, it is noteworthy that on completion of wound healing (POD 15), HPS-treated wounds generally showed lower Ki67 expression compared to NT, whereas HPS-10% showed the least expression (Figure 4). Based on this data, we hypothesize that the relative downregulation of Ki67 in HPS-treated wounds may indicate an earlier completion of wound healing processes and underlines the accelerated wound closure observed with HPS treatment. However, we expected to see a similar (i.e., low) level of Ki67 expression in the HPS-40% group as in the HPS-10% group. The higher Ki67 expression in the HPS-40% group may be accounted for by the greater concentration of inflammatory (MMP-9, IL-8, CXCL-16) or other stimulatory (VEGF, IGF-BP3, Prolactin) cytokines that may well maintain a prolonged state of active wound repair [18,23,25], despite an overall comparable level of macroscopic epithelialization. These mechanisms require, however, further investigation.

Masson Trichrome analysis of collagen fibers has been conducted in many wound healing studies to determine the orientation and quantification of collagen fibers [48,49,50]. We detected no excess deposition of collagen fibers in HPS-hydrogel treated wounds (Figure 5), indicating no hypertrophic scar production in our study. Furthermore, there were no apparent differences on POD 15 in skin layer structure compared to NT, including epidermal thickness and number of skin appendages. This finding again demonstrates the safety of HPS treatment which likely prevents overproduction of scar tissue and fibrosis. Yet, based on the current findings we expected to see faster collagen fiber production at earlier wound healing stages with HPS treatment. As discussed beforehand, a temporal histological investigation examining the rate of collagen deposition should be carried out in future studies.

It has to be mentioned that in this study, we tested only the combination of HPS and alginate hydrogel (AH), without testing each component individually. We have previously demonstrated that topical HPS application can be optimally realized through a hydrogel-matrix carrier [16,18], which is required as a vehicle for local delivery of HPS protein factors. Alginate is an anionic linear polysaccharide that is obtained from brown algae or bacteria and serves as a natural biopolymer [51]. Studies have confirmed a promotion of wound healing through effects of AH alone, which are dependent on wound cellular-debridement by macrophage activation, dissolution of necrotic/fibrotic tissue and wound rehydration [51]. These are properties of AH which lead to faster wound closure, as reported in several rat wound models [52,53,54]. Evidently, there must have been a certain compounding effect of AH in the observed HPS-hydrogel acceleration of wound closure. Nonetheless, solely AH-driven promotion of new blood vessel growth could not so far be shown in vitro [53] and in vivo [52], while its effects on lymphatic vessels have yet to be investigated. Despite the fact that a control group of AH-only could not be included in this pilot study, which is certainly a limitation of this work, we did see different effects of HPS-dosing (10% vs. 40% concentration) that can only be accounted for by a cumulative effect of HPS growth factor-induced responses, especially in terms of accelerated wound healing, lymphangiogenesis and cell metabolism. To put this into greater perspective, a non-hypoxia conditioned serum, PRP (activated by CaCl2), speeded up wound healing by only 0.5 days [44]. Therefore, HPS-hydrogel therapy is possibly 3 times more effective than PRP in this model. In terms of study design, we conducted this first animal experiment based on the ‘3-R principle’ (Reduction, Refinement, Replacement), to assess the utility of HPS as a potential wound healing therapy. By completing this pilot study, we could confirm the safety and effectiveness of topical HPS-hydrogel application in comparison to no treatment. Our findings encourage further investigation using a formal set of control groups, including a carrier-only group.

Another limitation of this study was the inability to use autologous murine blood, which was, unfortunately, not possible to draw from these mice, due to physiologically-insufficient blood volume to produce adequate HPS for testing. Our primary goal, however, focuses on autologous HPS application in humans, which can better suit the inter-individual variability of growth factor responses involved in the wound repair process than any allogeneic therapy. For these reasons, we used human blood-derived HPS, which was tested on immunocompromised mice in order to exclude possible allergic/autoimmune reactions that could have interfered with wound healing. The fact that the HPS secretome tested was cell-free may indeed have reduced this confounding factor.

In a further stage, the efficacy of HPS also remains to be determined in the setting of pathological (i.e., non-traumatic) wound healing, such as in diabetes and peripheral vascular disease. Previously conducted in vitro experiments showed no difference in terms of pro-angiogenic factor composition between HPS obtained from diabetic patients or patients receiving anticoagulants and HPS obtained from healthy blood donors [22]. This is promising for the clinical translation of this approach in this patient group, that is burdened by chronic leg ulcers, commonly necessitating partial or complete lower extremity amputation and complex reconstructive surgery [55]. Beyond reducing overall morbidity and the length of patient hospitalization, HPS holds, therefore, the potential to reduce the large healthcare costs associated with these conditions.

## 5. Conclusions

Our in vivo findings demonstrate the regenerative potential of blood-derived hypoxia preconditioned serum (HPS)-hydrogel on wound healing. The promising results of this study suggest that this therapeutic method could be similarly efficacious in settings where the physiological mechanisms of wound healing are impaired. The wider application of HPS-based treatments may have a true positive impact in reducing healthcare costs, although this should not be considered as a replacement treatment for standard medical care, such as thorough debridement of necrotized tissue, but rather as a complementary therapy for bioactively supporting tissue regeneration. Further in vivo studies should be conducted to confirm the safety and to analyze temporal molecular mechanisms of hypoxia preconditioned blood-derived secretomes in wound repair.

## 6. Patents

Device-based methods for localized delivery of cell-free carriers with stress-induced cellular factors. (AU2013214187 (B2); 9 February 2017): Schilling Arndt, Hadjipanayi Ektoras, Machens Hans-Günther.

## Abbreviations

ADSC	Adipose-derived stem cells
bFGF	Basic fibroblast growth factor
CD31	Cluster of differentiation 31
CXCL-16	Chemokine (C-X-C motif) ligand-16
DAB	Diaminobenzidine
EWS	Extracorporeal wound simulation
HPS	Hypoxia preconditioned serum
IGF-BP3	Insulin-like growth factor-binding protein 3
IL-8	Interleukin-8
LYVE-1	Lymphatic vessel endothelial hyaluronan receptor-1
MMP-9	Matrix metalloproteinase-9
MSC	Mesenchymal stem cell
NT	No treatment
PBC	Peripheral blood cells
PRP	Platelet rich plasma
POD	Post-operative day
PF-4	Platelet factor-4
TSP-1	Thrombospondin-1
VEGF	Vascular endothelial growth factor
